# Barriers to help-seeking for Malaysian women with symptoms of breast cancer: a mixed-methods, two-step cluster analysis

**DOI:** 10.1186/s12913-023-09046-x

**Published:** 2023-03-01

**Authors:** Nadia Rajaram, Maheswari Jaganathan, Kavitha Muniandy, Yamuna Rajoo, Hani Zainal, Norlia Rahim, Nurul Ain Tajudeen, Nur Hidayati Zainal, Azuddin Mohd Khairy, Mohamed Yusof Abdul Wahab, Soo Hwang Teo

**Affiliations:** 1 Cancer Research Malaysia, Subang Jaya, Malaysia; 2 Kementerian Kesihatan Malaysia (Hospital Tengku Ampuan Rahimah, Klang), Klang, Malaysia; 3 University Malaya Cancer Research Institute, Kuala Lumpur, Malaysia

**Keywords:** Help seeking, Early presentation, Breast cancer, Early detection, Barriers, Malaysia

## Abstract

**Background:**

Improving help-seeking behaviour is a key component of down-staging breast cancer and improving survival, but the specific challenges faced by low-income women in an Asian setting remain poorly characterized. Here, we determined the extent of help-seeking delay among Malaysian breast cancer patients who presented at late stages and explored sub-groups of women who may face specific barriers.

**Methods:**

Time to help-seeking was assessed in 303 women diagnosed with advanced breast cancer between January 2015 and March 2020 at a suburban tertiary hospital in Malaysia. Two-step cluster analysis was conducted to identify subgroups of women who share similar characteristics and barriers. Barriers to help-seeking were identified from nurse interviews and were analyzed using behavioural frameworks.

**Results:**

The average time to help-seeking was 65 days (IQR = 250 days), and up to 44.5% of women delayed by at least 3 months. Three equal-sized clusters emerged with good separation by time to help-seeking (*p* < 0.001). The most reported barrier across clusters was poor knowledge about breast health or breast cancer symptoms (36.3%), regardless of help-seeking behaviour (*p* = 0.931). Unexpectedly, women with no delay (9 days average) and great delay (259 days average) were more similar to each other than to women with mild delays (58 days average), but, women who experienced great delay reported poor motivation due to fear and embarrassment (*p* = 0.066) and a lack of social support (*p* = 0.374) to seek help.

**Conclusions:**

Down-staging of breast cancer in Malaysia will require a multi-pronged approach aimed at modifying culturally specific social and emotional barriers, eliminating misinformation, and instilling motivation to seek help for breast health for the women most vulnerable to help-seeking delays.

**Supplementary Information:**

The online version contains supplementary material available at 10.1186/s12913-023-09046-x.

## Background

Although breast cancer incidence is higher in high income countries (HICs), incidence rates have been rising steadily in the majority of low- or middle-income countries (LMICs) [1]. For example, in Asia, breast cancer incidence has risen by up to 6% annually between 1998 and 2012 [1]. Furthermore, data from 2000 to 2014 show that overall 5-year survival from breast cancer is lower in LMICs, averaging 65–85% compared to more than 90% in most HICs [2]. In Malaysia, breast cancer is the most commonly diagnosed cancer and almost half of all breast cancer cases (47.9%) are diagnosed at late stages (Stage 3 and 4) [3]. Despite fully or partially subsidized breast cancer screening services in government healthcare facilities in Malaysia, uptake of screening remains low, with less than 35% of women over 40 years old reporting a clinical breast examination or a mammogram in the past 2 years [4]. Taken together, these statistics highlight the need to develop effective national cancer control plans for resource-constrained healthcare systems [5, 6].

Lower survival rates are largely attributed to late presentation of breast cancer as well as lack of timely and adequate access to treatment [7, 8]. The WHO Framework for Cancer Control describes the patient journey in 3 actionable intervals, which includes the presentation interval (symptom awareness to first encounter with healthcare), the diagnosis interval (first encounter to confirmed diagnosis), and the treatment interval (diagnosis to treatment onset) [9]. Compared to the latter two intervals, the presentation interval may have the most influence on the stage at diagnosis, and delays in this interval could increase 5-year mortality risk by 12% [8]. The presentation interval is also most likely driven by modifiable patient factors, making it a possible target for intervention to down-stage breast cancer [10].

There is current extensive research on factors that are associated with delays in the presentation interval, or help-seeking delays, in both HICs and LMICs [10–12]. Overarchingly, common reasons for delay include poor health literacy or breast cancer awareness, fear, social influences, and socioeconomic barriers. Some themes appear to be more common in LMICs, such as health care access barriers, cancer stigma, and sociocultural factors including use of alternative or traditional treatments [10, 12]. Previous research among Malaysian women have identified similar barriers to early presentation, including poor knowledge about breast health, the preference for alternative treatments, as well as emotional and financial barriers [13–15]. Notably, much of the research on help-seeking delay have investigated these determinants or barriers as acting independently [11, 16], and few have used behavioural frameworks to tease out the complex and dynamic barriers that women face in seeking help for their breast symptoms [12].To address these gaps for Malaysian women, we sought to determine the average time to help-seeking for breast symptoms among Malaysian women presenting with Stage 3 and 4 breast cancer. Using established behavioural frameworks, we sought to identify the underlying barriers to help-seeking in this population and explored for subgroups of women who may face specific barriers.

## Methods

### Sample selection

As part of the Patient Navigation Programme [17], women with suspicious breast symptoms were referred to the Pink Ribbon Centre at a suburban tertiary hospital in Malaysia (Tengku Ampuan Rahimah Hospital, Klang) for diagnosis and treatment. A total of 371 newly diagnosed breast cancer patients presented to the centre with advanced disease (Stage 3 and 4) between January 2015 and March 2020 and were included in the analysis except if they died prior to the interview (*n* = 12), were not Malaysian (*n* = 18), or if there was missing data for any of the variables used in the analysis (*n* = 38). The final analysis included 303 patients.

### Data collection

Patients were interviewed at the Pink Ribbon Centre by a trained nurse responsible for their care. A structured questionnaire was used to collect socio-demographic status, medical history, breast cancer risk factors, risk management behaviour, and barriers to help-seeking. The questionnaire was developed by incorporating best practices in patient assessment tools used in breast cancer clinics as well as factors that are relevant to breast cancer risk and help-seeking behaviour among Malaysian women. It was tested in a pilot study of 30 breast cancer patients prior to data collection *(data not published)*. To ease in the analysis and interpretation of the results, some variables from the questionnaire were transformed into binary responses. Patients were categorized as knowing signs and symptoms of breast cancer if they correctly identified at least one sign or symptom of breast cancer. Similarly, patients were categorized as knowing risk factors of breast cancer if they were able to correctly list at least one known risk factor. Patients were reported to have a transportation barrier if they relied on public transport to attend hospital visits. Lastly, patients who were unable to speak the national language (Bahasa Malaysia) or English were categorized as having a language barrier.

Additionally, nurses used open-ended questions to capture more information about barriers and help-seeking behaviour in narrative form. This qualitative data is used in the thematic analysis.

### Statistical analysis

Standard descriptive statistics were used to describe the distribution of patients by demographic and socio-economic factors, knowledge, screening, barriers, and time to help-seeking. The interquartile range (IQR) for continuous variables was calculated by subtracting the 1^st^ quartile from the 3^rd^ quartile of the distribution. Time to help seeking was defined from the date of symptom awareness to the date of first medical encounter for the symptom, which was self-reported by patients in the structured questionnaire. If the day or month information was incomplete, we assumed it to be the 1^st^ of the month or June of that year, respectively.

A multivariable logistic regression model was used to assess the association between patient factors and time to help-seeking (≤ 3 months vs > 3 months) [8]. Next, two-step cluster analysis was performed on the dataset using the algorithm in the IBM® SPSS® Statistics software. Using backward elimination, variables were removed if they were poorly associated with time to help-seeking in the regression model (lenient *p* > 0.600) or showed poor predictor importance in two-step cluster analysis (< 15%, Supplementary Table 1). The model was finalized when suitable clustering parameters were achieved (Supplementary Table 1). Internal validation of the cluster analysis was conducted using principal component analysis (Supplementary Fig. 1). The clusters were labelled based on the average time to help-seeking within the cluster, i.e. “no delay”, “mild delay”, and “great delay”. The “great delay” cluster corresponds to a delay of more than 3 months, which is associated with poorer prognosis and survival from breast cancer [8].

Differences across clusters were assessed with global Kruskal–Wallis tests for non-parametric continuous variables and independent Chi-square tests for homogeneity for categorical variables. Variables with *p* < 0.100 in global tests were subjected to pairwise Wilcoxon tests with Benjamini–Hochberg correction or Chi-square tests with Bonferroni correction, respectively. These analyses were performed using the R Statistical environment (v4.0.3).

### Thematic analysis

Barriers to help-seeking were identified from notes collated by the nurse during interview with patients. These barriers were grouped into themes and further mapped to a combined matrix of two behavioural models, namely the Capability, Opportunity, and Motivation Model for Behaviour (COM-B) and the Theoretical Domains Framework (TDF) [18]. The TDF is a compilation of behavioural frameworks that seeks to provide a comprehensive, practical guideline for implementation research, including health services utilization research [18]. This process was conducted independently by two reviewers and discrepancies were resolved by consensus. A summary of the barriers in each COM-B/TDF domain is described (Supplementary Table 2). Fisher’s Exact tests were used to test for differences in the distribution of COM-B/TDF domains across clusters. These analyses were performed using the R Statistical environment (v4.0.3).

All hypotheses were two-sided, and *p* < 0.05 was considered statistically significant.

## Results

### Cohort description and time to help seeking

**Fig. 1:**
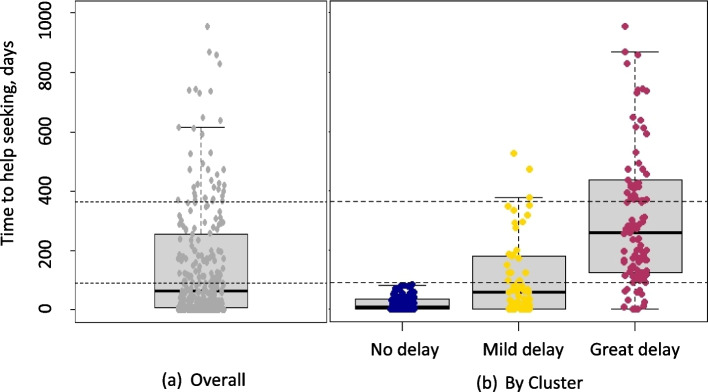
Distribution of time to help seeking **a** overall and **b** by cluster

In this cross-sectional analysis of 303 newly diagnosed Malaysian breast cancer patients presenting with advanced disease, the average time to help-seeking was approximately 2 months (median = 65 days, IQR = 250 days). Almost half of women (*n* = 136, 44.5%) delayed help-seeking by > 3 months. Also, as shown in Fig. 1, there was great variation in time to help-seeking in the overall cohort, with 18% of the patients reporting > 1 year delay.

A low proportion of women reported knowing the signs and symptoms (42.6%) or risk factors (16.5%) of breast cancer (Table 1). In this cohort, only 17.8% reporting ever being screened for breast cancer. With regards to help-seeking behaviour, most women (64.4%) reported that their health decisions are made jointly with their families, whilst 13% of women reported that they make their own health decisions. For up to 21% of women, however, their health decisions are made by their family.

**Table 1:** Demographics of breast cancer patients, by time to help-seeking

		**By time to help-seeking**			
**Characteristics**	Overall	≤ 3 months	> 3 months	**Association with delay in help seeking (> 3 months)**	
	(*n* = 303)	(*n* = 167)	(*n* = 136)	OR [95%CI]	*p*-value
**Demographic factors**					
Age, years, median (IQR)	55.1 (14.0)	54.5 (14.2)	55.9 (13.8)	1.01 [0.98,1.05]	0.455
Ethnicity, n(%)					
Malay	179(59.1)	90(53.9)	89(65.4)	Ref	
Indian	77(25.4)	50(29.9)	27(19.9)	1.03 [0.39,2.69]	0.226
Chinese	47(15.5)	27(16.2)	20(14.7)	0.55 [0.21,1.42]	0.957
Marital status, n(%)					
Married	181(59.7)	103(61.7)	78(57.4)	Ref	
Divorced/widowed	86(28.4)	41(24.6)	45(33.1)	1.55 [0.63,3.82]	0.340
Single	35(11.6)	22(13.2)	13(9.6)	0.70 [0.21,2.16]	0.535
Children, n(%)					
None	50(16.5)	30(18.0)	20(14.7)	^b^	
1–3	145(47.9)	76(45.5)	69(50.7)		
> 3	107(35.5)	61(36.5)	46(33.8)		
**Socio-economic factors**					
Currently employed, n(%)					
No	220(72.6)	121(72.5)	99(72.8)	Ref	
Yes	77(25.4)	42(25.1)	35(25.7)	1.19 [0.50,2.84]	0.686
Household income, n(%)					
≥ RM800/month	205(67.7)	115(68.9)	90(66.2)	Ref	
< RM800/month	89(29.4)	49(29.3)	40(29.4)	1.44 [0.64,3.27]	0.374
Formal education, n(%)					
Incomplete	180(59.4)	103(61.7)	77(56.6)	Ref	
Secondary/tertiary	120(39.6)	61(36.5)	59(43.4)	1.31 [0.56,3.02]	0.532
**Knowledge & screening**^a^					
Signs & symptoms, n(%)	129(42.6)	61(36.5)	68(50.0)	2.03 [0.91,4.60]	*0.085*
Risk factors, n(%)	50(16.5)	21(12.6)	29(21.3)	1.21 [0.47,3.12]	0.694
Ever screened, n(%)	54(17.8)	28(16.8)	26(19.1)	1.38 [-0.53,3.64]	0.507
**Barriers**					
Distance to hospital, n(%)					
< 10 km	172(56.8)	99(59.3)	73 (53.7)	Ref	
≥ 10 km	131(43.2)	68(40.7)	63 (46.3)	1.34 [0.68,2.64]	0.394
Transportation barrier^a^, n(%)	81(26.7)	45(26.9)	36 (26.5)	0.89 [0.40, 1.97]	0.783
Language barrier^a^, n(%)	21(6.9)	11(6.6)	10 (7.4)	1.46 [0.42,5.12]	0.547
Decision making, n(%)					
Self	41(13.5)	23(13.8)	18(13.2)	Ref	
With family	195(64.4)	106(63.5)	89(65.4)	1.04 [0.39,2.78]	0.936
By family	63(20.8)	37(22.2)	26(19.1)	1.22 [0.41,3.65]	0.724
Bad experience/crisis^a^, n(%)	87(28.7)	41(24.6)	46(33.8)	1.15 [0.53,2.49]	0.720

^a^Reference group in multivariable logistic regression = “No”

^b^Number of children was highly correlated with marital status (VIF > 3) and was therefore excluded from multivariable analysis

Furthermore, nearly one-third (29%) of women reported a past bad experience with healthcare or a life crisis. Up to 43% of women lived more than 10 km from the hospital and 26.7% reported transportation as a barrier to seeking healthcare services. A small minority reported a language barrier when communicating with hospital staff (6.9%).

We observed no significant differences when comparing the characteristics of women by time to help-seeking (≤ 3 months vs > 3 months, Table 1).

### Distribution of patients by two-step cluster analysis grouping

Given the lack of association in multivariable logistic regression when comparing women who sought help within 3 months versus after 3 months of symptom recognition, we conducted a two-step cluster analysis to explore for groups of women who may share similar characteristics and experiences, and whether certain groups were more likely to delay help-seeking. It revealed three distinct clusters with good separation by time to help-seeking (*p* < 0.001, Fig. 1). The average time to help-seeking was 9 days (Interquartile range, IQR = 32.8) for women in the “No delay” cluster, 58 days (IQR = 180 days) in the “Mild delay” cluster, and 259 days in the “Great delay” cluster (IQR = 314 days). In the “Great delay” cluster, up to 90% of women experienced delays of > 3 months.

Unexpectedly, we observed many similarities between women with no delay and women with great delay, while women with mild delay in help-seeking were characteristically different (Table 2). Compared to women with no delay and great delay, women with mild delay were older (67 vs 47 and 52 years old, respectively, *p* < 0.001) and were less likely to be Malay (38.3% vs 63.8% and 75.2%, *p* < 0.001). Women with mild delay also reported more socioeconomic barriers, such as low education, unemployment, and low household income (*p* < 0.001, respectively). Furthermore, they were less likely to report knowing about signs and symptoms or risk factors of breast cancer (*p* < 0.001, respectively).

**Table 2:** Distribution of breast cancer patients, by two-step cluster analysis grouping

	**Distribution by cluster**^**‡**^				**Pairwise comparison,** ***p*-value**		
**Characteristics**	No delay (*n* = 94)	Mild delay (*n* = 81)	Great delay (*n* = 113)	p_global_	No vs Mild	Mild vs Great	No vs Great
**Time to help seeking**							
In days^a^, median (IQR)	9 ± 33	58 ± 180	259 ± 314	** < 0.001**			
> 3 months delay, n(%)	0(0)	29(35.8)	101(89.4)	** < 0.001**	***	***	***
**Demographic factors**							
Age in years^a^, n(%)	47.5 ± 16.8	67.0 ± 17.0	52.0 ± 13.0	** < 0.001**			
Ethnicity^a^, n(%)							
Malay	60(63.8)	31(38.3)	85(75.2)	** < 0.001**	*	***	
Indian	24(25.5)	27(33.3)	20(17.7)				
Chinese	10(10.6)	23(28.4)	8(7.1)				
Marital status^a^, n(%)							
Married	85(90.4)	23(28.4)	66(58.4)	** < 0.001**	***	***	
Divorced/Widowed	2(2.1)	47(58.0)	32(28.3)		***		***
Single	7(7.4)	11(13.6)	15(13.3)				
No. of children, n(%)							
None	14(14.9)	15(18.5)	19(16.8)	**0.043**			
1–3	51(54.3)	27(33.3)	58(51.3)			**	
> 3	29(30.9)	39(48.1)	35(31.0)				
**Socio-economic factors**							
Currently employed, n(%)	30(31.9)	8(9.9)	37(32.7)	** < 0.001**	**	***	
Household income, n(%)							
≥ RM800/month	92(97.9)	8(9.9)	102(90.3)	** < 0.001**	***	***	
< RM800/month	2(2.1)	73(90.1)	11(9.7)		***	***	
Formal education^a^, n(%)							
Incomplete	47(50.0)	75(92.6)	48(42.5)	** < 0.001**			
Secondary/tertiary	47(50.0)	6(7.4)	65(57.5)		***	***	
**Knowledge & screening**							
Know sign/symptoms^a^, n(%)	47(50.0)	12(14.8)	67(59.3)	** < 0.001**	***	***	
Know risk factors, n(%)	16(17.0)	2(2.5)	30(26.5)	** < 0.001**	**	***	
Ever screened, n(%)	20(21.3)	5(6.2)	26(23.0)	**0.006**	**	**	
**Barriers**							
Distance to hospital^a^, n(%)							
< 10 km	50(53.2)	53(65.4)	61(54.0)	0.190			
≥ 10 km	44(46.8)	28(34.6)	52(46.0)				
Transportation barrier, n(%)	24(25.5)	29(35.8)	25(22.1)	*0.062*			
Language barrier, n(%)	3(3.2)	9(11.1)	6(5.3)	*0.076*			
Decision making, n(%)							
Self	13(13.8)	11(13.6)	15(13.3)	0.340			
With family	64(68.1)	46(56.8)	76(67.3)				
By family	17(18.1)	23(28.4)	19(16.8)				
Bad experience/crisis^a^, n(%)	12(12.8)	31(38.3)	43(38.1)	** < 0.001**	*		***

^‡^A total of 15 women (5%) were not clustered due to missing data. Pair-wise *p*-values

^***^p-value > 0.001

** *p*-value > 0.05

*p-value > 0.1

^a^Variables included in the two-step cluster analysis model

Interestingly, we found that marital status and past bad experiences may be associated with delayed help-seeking behaviour in this cohort (Table 2).

Compared to women with no delay, women with mild and great delay were more likely to be divorced or widowed (58.0% and 28.3% vs 2.1% among no delays, *p* < 0.001). Up to 38% of women in the two delay clusters reported a past bad experience in healthcare or a life crisis, compared to only 12.8% among women with no delays (*p* < 0.001).

### Barriers to help seeking among Malaysian women

Using theoretical frameworks, we mapped the barriers to help-seeking for Malaysian women presenting with advanced breast cancer into a matrix of 3 COM-B domains and 10 TDF domains (Table 3). Lack of knowledge, in the capability domain, was the most common barrier to help-seeking (36.3%). Next, barriers were commonly reported in the motivation domain, such as emotional barriers (22.1%) and optimism (20.8%). Approximately 11% of patients report lack of opportunity to seek help, due to physical and social barriers.

**Table 3:** Distribution of barriers, mapped to the COM-B and TDF domains, overall and by cluster

		**Distribution by cluster,** **n(%)**			
**Domain** (COM-B sub-domain/*TDF*)	**Overall** (*n* = 303)	No delay (*n* = 94)	Mild delay (*n* = 81)	Great delay (*n* = 113)	p_global_
**Capability**					
Psychological/K*nowledge*	110(36.3)	32(34.0)	30(37.0)	41(36.3)	0.931
Physical/*Environmental context*	18(5.9)	5(5.3)	6(7.4)	5(4.4)	0.668
Physical/*Skills*	4(1.3)	0(0)	3(3.7)	0(0)	**0.021**^†^
**Motivation**					
Automatic/*Emotion*	67(22.1)	14(14.9)	21(25.9)	32(28.3)	*0.066*
Reflective/*Optimism*	63(20.8)	21(22.3)	12(14.8)	26(23.0)	0.319
Reflective/*Belief about consequence*	26(8.6)	9(9.6)	2(2.5)	14(12.4)	**0.050**^‡^
**Opportunity**					
Physical/*Environmental context*	35(11.6)	12(12.8)	12(14.8)	10(8.8)	0.416
Social/*Social identity*	34(11.2)	12(12.8)	8(9.9)	13(11.5)	0.823
Social/*Social influences*	15(5.0)	3(3.2)	4(4.9)	7(6.2)	0.616
Social/*Environmental context*	14(4.6)	3(3.2)	3(3.7)	8(7.1)	0.374

^†^Unable to compute pair-wise comparison due to zero values

^‡^*p*-value > 0.05 comparing mild vs great delay, all other pair-wise comparisons were not statistically significant

We compared the distribution of barriers by time to help-seeking (Supplementary Table 3). Compared to women who sought help within 3 months, women who delayed help-seeking were more likely to report lack of motivation, including emotional barriers (25.7% vs 19.2%, *p* = 0.239), optimism (24.3% vs 18.0%, *p* = 0.251), and belief in consequences (10.3% vs 7.2%, *p* = 0.470). Social influences were also more commonly reported among women who delayed help-seeking (7.4% vs 3.0%, *p* = 0.147). However, these associations were not statistically significant.

When comparing the distribution of barriers by clusters, on the other hand, some similarities were observed among women with no delay and great delays (Table 3). Between 9–12% of women in these clusters reported belief in consequences as a barrier to help-seeking, compared to 2.5% among women with mild delay (*p* = 0.050). Similarly, women with no or great delay were more likely to report optimism as a reason for delayed help-seeking but this was not significantly different from women with mild delay (22–23% vs 14.8%, *p* = 0.319). The results observed here for women with no delay contrasts the analysis by time to help-seeking in Supplementary Table 3.

Instead, emotional and social barriers appear to be important facilitators for delayed help-seeking behaviour in this cohort. More than 25% of women with mild and great delay reported emotional barriers, compared to only 15% of women with no delay (*p* = 0.066). Furthermore, 7% of women with the great delay reported barriers in their social environment, compared to 3–4% among women with no or mild delay, but this difference was small and not statistically significant (*p* = 0.374). Notably, women with physical capability barriers were all clustered within the mild delay group (3.7%).

## Discussion

In this study of 303 Malaysian women presenting with advanced breast cancer, the average time from symptom discovery to help-seeking was approximately 2 months, similar to previous reports in LMICs [12]. Here, up to 45% of women experienced help-seeking delays by more than 3 months, and 18% were delayed by more than 1 year. Poor knowledge about breast cancer was commonly and consistently reported in this cohort of women, regardless of help-seeking behaviour. We found that women could be clustered into 3 groups based on their help-seeking behaviour. Intriguingly, instead of a graduated effect, we found that women with least and greatest delay were more similar to each other compared to women with mild delay. Importantly, we found that emotional and social barriers most likely facilitated help-seeking delay in this cohort. This study highlights that Malaysian women who face delays in seeking help are not a homogeneous group, and that varied solutions are required to effectively improve early detection of breast cancer in Malaysia.

Our study shows that poor cancer awareness remains an important barrier for Malaysian women. This is consistent with other studies that have highlighted knowledge as a primary barrier to help-seeking behaviour in both HICs and LMICs, including in Malaysia and Singapore, where women who were more aware about breast health were more likely to seek help early [11, 12, 15, 19]. As a result, the public health response to increasing early detection of breast cancer has centred on campaigns that create and raise awareness. However, the long-term efficacy of such campaigns in reducing presentation delay, and ultimately in down-staging breast cancer, is not well-studied [20]. Notably, our results suggest that addressing poor knowledge alone is likely insufficient to improve early detection of breast cancer.

Instead, addressing emotional barriers may empower women to seek help early for their breast symptoms. Previous reports suggest that emotional messaging in information about breast health awareness is an important driver for help-seeking behaviour [21]. Fear is a commonly used emotion in public health campaigns, including for breast cancer awareness, but its effect on help-seeking behaviour is inconsistent across populations [22]. While some studies show that fear has a positive influence [22], we show that fear and embarrassment were important deterrents to help-seeking. This is consistent with other studies of Asian women, where fear is often reported as one of the main reasons for late presentation [15, 23]. Alternatively, emotional messaging with positive reinforcements that reduce the fear of treatment and death may serve as stronger motivation for Asian women and is an area of pressing unmet need that requires further investigation.

Additionally, we show that a poor social environment may preclude women of the opportunity to seek help. This finding is comparable to past studies of Asian women, where poor social support and negative social influences are often reported as primary barriers to seeking help for breast cancer symptoms [14, 15, 24], and may be a more significant barrier than lack of knowledge in this region [13]. In Asian communities, family members appear to have both direct and indirect roles in a women’s decision to seek help [19, 25, 26]. A supportive family can be an enabler of early help-seeking behavior and adherence to treatment [19], whereas lack of support or unstable family dynamics often lead to confusion, uncertainty, and ultimately, delay in seeking medical treatment [25]. Therefore, interventions to improve screening and early detection that consider family dynamics and socio-cultural factors may prove to be more effective than a one-size-fits-all campaign [27].

Surprisingly, in our cohort, women with no delay observed similar characteristics and barriers to those with great delay. For example, we observed that women with no delay and great delay similarly reported the use of alternative treatment prior to seeking medical attention. The preference for alternative treatment is commonly reported in this region [13, 14, 19] as well as other LMICs [10], and has been previously shown to deter help-seeking behaviour [25]. Here, we suggest that use of alternative treatment may not always lead to delays in help-seeking. Instead, delay occurs when there are other emotional and social pressures that reinforces a women’s belief that medical care is not necessary, not accessible, or not sufficient to treat her symptoms [15]. This analysis further illustrates the diversity of women and the challenges that they face in seeking help for breast symptoms, which may not be elucidated in studies where barriers are assumed to act independently.

To our knowledge, this is the first study to use a mixed-method cluster analysis to understand barriers to help-seeking behavior for breast cancer symptoms. It considers the possibility that women delay help-seeking by different degrees and for various and multiple reasons [11, 15]. Coupling the two-step cluster analysis with thematic analysis has enabled us to study the intersections between patient characteristics and the barriers faced by different groups of women in the population. Specifically, it has identified emotional coping as the main difference between women who present with no or mild delay compared to women who have great delay, raising the intriguing possibility that public campaigns that focus on positive messaging about survival of cancer and emotional coping could have a more positive impact on help-seeking behavior than those which focus on knowledge of signs and symptoms alone.

This study is not without limitations. Firstly, we have only examined patient factors and have not considered healthcare factors, such as access and coordination of care [12]. Secondly, the women studied here are advanced breast cancer patients diagnosed at a suburban tertiary hospital in Malaysia. Therefore, the findings may not be inferred to all Malaysian breast cancer patients. Furthermore, patients who presented early in our study may represent a unique group of women with more aggressive disease and may not be comparable to women who present with early-stage breast cancer. Thirdly, time to help-seeking was determined based on self-reported dates from patients, which might be subject to recall bias and social desirability bias, which could have led to misclassification. Lastly, the data used for the thematic analysis represent patient barriers identified by nurses during interviews with patients, rather than patient-reported barriers. Future research could explore whether there are differences between nurse-perceived and patient-reported barriers. It is also important to note that the questionnaire used was not tested for validity and reliability, which could have led to some variability in the collected responses.

## Conclusions

Whilst poor breast cancer knowledge and awareness was commonly reported among Malaysian women, we show that women face diverse and intersecting barriers when seeking help for breast symptoms which cannot be addressed by traditional breast cancer awareness campaigns alone. Instead, public health strategies that seek to reduce help-seeking delays and down-stage breast cancer in Malaysia should incorporate targeted emotional messaging with positive reinforcements that consider local family dynamics and socio-cultural factors.

## Supplementary Information

**Additional file 1: Supplementary Table 1.** Model selection for two-step cluster analysis. **Supplementary Table 2.** Description of themes according to COM-B and TDF domains. **Supplementary Table 3.** Distribution of barriers, mapped to the COM-B and TDF domains, by time to help seeking. **Supplementary Figure 1.** Internal validation of clustering using principal component analysis (PCA) showing (a) separation plot labelled with two-step cluster analysis groups, and (b) relative contribution of each variable to separation.

## Data Availability

The datasets generated and/or analysed during the current study are not publicly available due privacy or ethical restrictions but are available from the corresponding author on reasonable request.

## Abbreviations

COM-B: Capability, Opportunity, and Motivation Model for Behaviour
HIC: High income countries
IQR: Interquartile range
LMIC: Low- or middle-income countries
TDF: Theoretical Domains Framework
